# Efficacy of the Video-feedback Intervention to promote Positive Parenting and Sensitive Discipline in Twin Families (VIPP-Twins): Study protocol for a randomized controlled trial

**DOI:** 10.1186/s40359-016-0139-y

**Published:** 2016-06-06

**Authors:** Saskia Euser, Marian J. Bakermans-Kranenburg, Bianca G. van den Bulk, Mariëlle Linting, Rani C. Damsteegt, Claudia I. Vrijhof, Ilse C. van Wijk, Eveline A. Crone, Marinus H. van IJzendoorn

**Affiliations:** Centre for Child and Family Studies, Leiden University, P.O. Box 9555, Leiden, 2300 RB Netherlands; Leiden Consortium on Individual Development, Leiden University, P.O. Box 9555, Leiden, 2300 RB Netherlands; Leiden Institute for Brain and Cognition, P.O. Box 9600, Leiden, 2300 RC Netherlands; Institute of Psychology, Brain and Development Lab, Leiden University, P.O. Box 9555, Leiden, 2300 RB Netherlands

**Keywords:** Intervention, RCT, Parenting, Behavioral control, Social competence, Differential susceptibility

## Abstract

**Background:**

Intervention programs with the aim of enhancing parenting quality have been found to be differentially effective in decreasing negative child outcomes such as externalizing behavioral problems, resulting in modest overall effect sizes. Here we present the protocol for a randomized controlled trial to examine the efficacy of the Video-feedback Intervention to promote Positive Parenting and Sensitive Discipline for Twin Families (VIPP-Twins) on parenting quality and children’s behavioral control and social competence. In addition, we aim to test the differential susceptibility theory; we examine differential efficacy of the intervention based on genetic make-up or temperament for both parents and children. Lastly, we explore neurobiological mechanisms underlying intervention effects on children’s developmental outcomes.

**Methods/design:**

The original VIPP-SD was adapted for use in families with twins. The VIPP-Twins consists of five biweekly sessions in which the families are visited at home, parent-child interactions are videotaped and parents receive positive feedback on selected video fragments. Families (*N* = 225) with a same sex twin (mean age = 3.6 years) were recruited to participate in the study. The study consists of four assessments. After two baseline assessments in year 1 and year 2, a random 40 % of the sample will receive the VIPP-Twins program. The first post-test assessment will be carried out one month after the intervention and there will be a long term follow-up assessment two years after the intervention. Measures include observational assessments of parenting and children’s social competence and behavioral control, and neurobiological assessments (i.e., hormonal functioning and neural (re-)activity).

**Discussion:**

Results of the study will provide insights in the efficacy of the VIPP-Twins and reveal moderators and mediators of program efficacy. Overall the randomized controlled trial is an experimental test of the differential susceptibility theory.

**Trial registration:**

Dutch Trial Register: NTR5312; Date registered: July 20, 2015.

**Electronic supplementary material:**

The online version of this article (doi:10.1186/s40359-016-0139-y) contains supplementary material, which is available to authorized users.

## Background

Parenting quality affects a wide array of social, emotional and cognitive child outcomes (e.g., [1, 2]), and intervention programs with the aim of enhancing parenting quality have been found to be effective in decreasing negative child outcomes such as externalizing behavioral problems (e.g., [3, 4]). However, intervention programs may not be equally effective for all families. Such individual differences between individuals in intervention efficacy result in modest overall effect sizes, as have been found for parenting intervention programs [5, 6]. The intervention effects on susceptible families may remain hidden when only overall effects are taken into account. In the current randomized controlled trial (RCT) we examine the efficacy of the Video feedback Intervention to promote Positive Parenting and Sensitive Discipline for Twin Families (VIPP-Twins) on parenting quality and children’s behavioral control and social competence. In addition, we examine markers of differential susceptibility that may be characterizing parents and children who are most open to the positive influences of the intervention program, and on possible neurobiological mechanisms of intervention effects on children’s developmental outcomes.

### Differential intervention effects

Differences in intervention efficacy may be explained by the differential susceptibility model [6–8]. According to this model, not all individuals are equally affected by their environment, and this difference in susceptibility is for better and for worse: Some individuals are more susceptible to both the adverse effects of negative environments and to the positive effects of a supportive environment than others. Moderators of the environmental effects are referred to as susceptibility markers. In the context of parenting, children’s dopamine related gene polymorphisms have been found to be susceptibility factors. For example, Knafo, Israel & Ebstein [9] found a relation between positive parenting and children’s prosocial behavior, but only for children with the DRD4 7-repeat allele. Children with the 7-repeat allele showed the most prosocial behavior when in a positive parenting environment, whereas children with the 7-repeat allele experiencing less positive parenting showed the least prosocial behavior. In addition, evidence for children’s differential susceptibility to a parenting intervention dependent on their genetic make-up was found in a randomized controlled trial [10]: Only children with the DRD4 7-repeat allele showed decreased daily cortisol production and decreased externalizing behavior at two years follow-up.

Kim and Kochanska [11] reported differential susceptibility to parenting with children’s temperament, or negative emotionality, as susceptibility marker. Infants with high negative emotionality developed the highest levels of effortful control and self-regulatory compliance toward their mothers in positive mother-child relationships, but the lowest levels in negative mother-child relationships. Mother-child relationship quality did not affect effortful control of self-regulation for children with low negative emotionality. Similar results were found for childcare quality as environmental factor and behavioral problems as outcome [12]; children with high negative emotionality were more affected by childcare quality, for better and for worse. Gene-by-environment interactions may also be involved in environmental effects on parenting. Parents with so called susceptibility genes were found to be less sensitive in the case of negative environments characterized by early childhood maltreatment, depression and/or daily (parenting) stressors, whereas they displayed the highest levels of sensitivity in positive, supportive environments [13, 14].

An important limitation of these studies is their correlational design. In correlational studies, the environment and the susceptibility marker may be correlated; children’s genetic make-up and their parenting environment may at least partly be caused by the same underlying factor, their parents’ genes [15]. Such gene-environment correlations make it impossible to examine the true moderating effect of heritable child characteristics in the relation between parenting environment and child outcomes. In the current study, we break the gene-environment correlation by using a randomized controlled trial with experimental manipulation of the parenting environment. Experimental manipulation is supposed to lead to positive changes in the family interactions and relationships, in particular in those parents and children who are more susceptible to the environment and profit more from the intervention whereas more susceptible individuals in control group families experiencing (mild) setbacks, conflicts or adversities may suffer more than their less susceptible counterparts.

### Causal mechanisms for change

Most intervention studies have examined the effect on child outcomes of a change for the better in the environment without addressing the question how improvement of the child’s environment results in more adaptive development. To unravel the mechanisms of change, we need to study intervention effects at different levels of functioning [16]. For example, parenting interventions may result in a more normative cortisol regulation over the day in children with negative childhood experiences such as parental separation or child maltreatment [17, 18], or in children with elevated levels of externalizing behavior symptoms [10]. Such neurobiological changes as a consequence of the changed environment might for example explain the persistence of intervention effects over time. In the current RCT, we examine changes at the behavioral, hormonal, and neural level to test for causal mechanisms underlying children’s more adaptive behavioral control and increased social competence after an intervention aimed at increasing supportive parenting behavior.

### VIPP-Twins

The Video-feedback Intervention to promote Positive Parenting and Sensitive Discipline (VIPP-SD) is an attachment based intervention that aims at enhancing parental sensitivity and sensitive discipline [19]. Previous randomized controlled trials have indicated the efficacy of the intervention program in a variety of samples and countries (for an overview, see [20]). A recent meta-analysis indicated an overall combined effect size of d = 0.47 on parental sensitivity [20]. VIPP-SD enhances parental sensitivity, decreases children’s insecure and disorganized attachment, and reduces children’s internalizing and externalizing behavior problems. The VIPP-SD has also been found effective in home-based and center-based child care (VIPP-CC; [21, 22]). In contrast to the VIPP-SD in family settings, the VIPP-CC focusses on sensitive caregiving behavior towards multiple children at the same time. The RCTs in child care indicated increased caregiver sensitivity and more positive caregiver attitudes towards sensitive caregiving and limit setting.

For the current study, we adapted the VIPP-SD protocol for use in families with twins (VIPP-Twins). Parents with twins are an important target group for parenting interventions, because compared to parents of singletons they have additional parenting difficulties. Higher financial and medical stressors and greater parenting demands, such as dividing their attention between two same aged children, put parents of twins at increased risk for mental health problems (see [23] for a review). Furthermore, as the parent is the target of the intervention, we will be able to examine any differential intervention effect between siblings within the same family across pre- to posttest, dependent on genetic dissimilarity in particular of the dopamine-related genetic pathways or in temperamental reactivity. Lastly, the inclusion of monozygotic and dizygotic twins will provide the possibility of genetic modeling of intervention effects.

### Aims and hypotheses

**Fig. 1:**
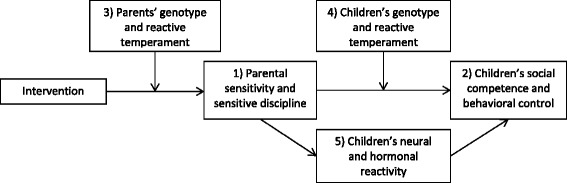
Overview of the most important outcomes measures, moderators and mediators of the study. *Note.* The numbers of the variables refer to the different aims of the study

1) The primary aim of the Leiden Consortium on Individual Development (L-CID) is to study the effect of the VIPP-Twins on parental sensitivity and sensitive discipline of the primary parent, one month after the intervention and two years after the intervention (see Fig. 1 for an overview of the different aims). It is expected that sensitivity and sensitive discipline of parents in the intervention condition will significantly increase post-intervention, compared to sensitivity and sensitive discipline of parents in the control condition, who have a similar number of (‘dummy’) contacts with the interveners. 2) The secondary aim is to examine the efficacy of the intervention in enhancing children’s levels of behavioral control and social competence, through increased parental sensitivity and sensitive discipline. 3) A tertiary aim is to test what works for whom by testing the differential susceptibility theory using temperamental reactivity and dopamine-related genotype as main susceptibility markers. We will examine whether the intervention effects on parental sensitivity and sensitive discipline are moderated by parents’ sensory sensitivity [24]) and genotype (dopamine related genetic pathways). 4) More exploratory, we will examine whether the intervention effect on children’s behavioral control and social competence is moderated by children’s genotype (dopamine related pathways) and/or by their reactive temperament, two markers of differential susceptibility that might overlap only partially and thus may have independent moderator effects. 5) Moreover, we aim to explore mediators of change in child outcomes by examining the intervention effect on changes in children’s neural activation and hormonal reactivity, and whether these differences mediate the observed changes in children’s behavioral outcomes.

**Fig. 2:**
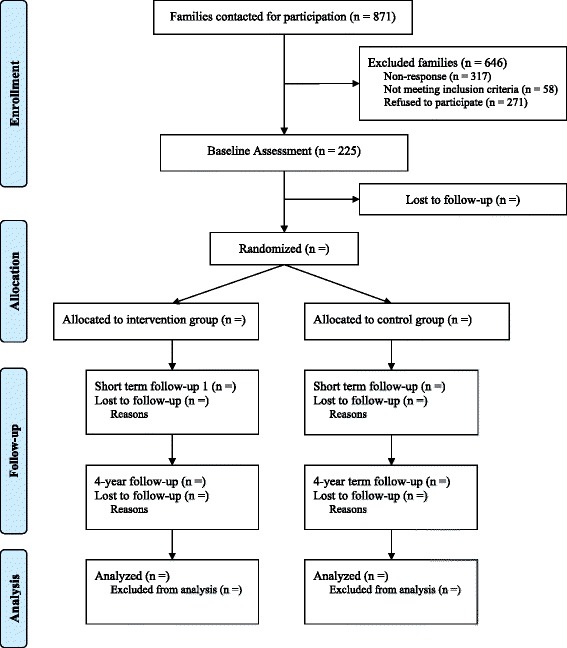
Flow chart of the phases through the randomized trial

## Methods/design

### Study design

The L-CID preschooler project is a 4-year randomized controlled trial. Participants are families with twins living in the western region of the Netherlands. The study consists of four assessments. Each assessment consists of a home or laboratory visit and several ambulatory assessments that are carried out by the parents at home. After two baseline assessments in year 1 and year 2, a random 40 % of the sample will receive an intervention aimed at enhancing parental sensitivity and sensitive discipline strategies of the primary caregiver, the VIPP-SD [19]. The first post-test assessment will be carried out one month after the intervention and there will be a long term follow-up two years after the intervention. This protocol paper adheres to the SPIRIT guidelines (see Additional file 1).

### Participants

#### Recruitment

Families with twins living in the western region of the Netherlands were selected from municipality records. Twins were eligible for participation if they had the same gender, if their parents were fluent in Dutch and if their parents and grandparents were born in Europe. Children with a congenital disability, psychological disorder, chronic illness, hereditary disease, or a visual or hearing impairment were excluded if the disorder will likely disable the child from performing the behavioral tasks or participating in the intervention. Also, children with a previously diagnosed intellectual disability (IQ < 70) were excluded from participation.

Eligible families (*n* = 871) received an invitation letter and information brochure by mail. Parents who were willing to participate, received a phone call during which a research assistant checked the inclusion criteria and provided additional information about the study. Families who met the inclusion criteria were then invited for the first home visit. To promote participant retention for the follow-up measures, parents will receive a financial reimbursement of €60 after home visits and €80 after laboratory visits, children will receive annual gifts and travel expenses will be compensated. In addition, participants will be informed about trial results in biannual newsletters.

#### Study sample

In total, 871 families received an invitation letter. A third of the families (37 %) did not respond to the invitation letter, 58 families (7 %) did not meet the inclusion criteria, and 271 families (31 %) did not want to participate. A final sample of 225 families (26 %) was enrolled in the study. Reasons for exclusion are shown in a flow chart (see Fig. 1). We received background information from 23 % of the eligible non-participating families. Sample characteristics from participating families and non-participating families who met the inclusion criteria are shown in Table 1. Participating families did not differ significantly from declining families on any of the background characteristics. At the time of recruitment, participating twins were on average 3.6 years old (SD = 0.57), and 50 % were boys.

**Table 1:** Demographic characteristics of participating and non-participating families

	Participating families (*n* = 225)	Non-participating, eligible families (*n* = 131)	
Twin characteristics			
Age at recruitment M (SD)	3.60 (0.58)	3.56 (0.53)	*p* = .45
Gender (% boys)	49.8	55.0	*p* = .35
Country of birth (% Netherlands)	99.6	100.0	*p* = .45
Family characteristics			
Primary parent (%)^1^			*p* = .97
Biological mother	92.0	86.4	
Biological father	8.0	7.6	
Age primary parent M (SD)	36.81 (4.58)	37.29 (4.51)	*p* = .39
Age second parent M (SD)	38.45 (5.65)	39.52 (4.78)	*p* = .11
Country of birth (% Netherlands)			
Primary parent	95.1	92.8	*p* = .39
Second parent	96.0	92.8	*p* = .21
SES – based on parents educational level (%)			*p* = .18
Low	7.1	6.1	
Medium	39.6	49.6	
High	53.3	44.3	
Number of children in the family M (SD)	2.82 (0.78)	2.90 (1.00)	*p* = .41
Primary parents’ marital status (%)			*p* = .25
Married or registered partnership	69.2	78.6	
Cohabiting	26.8	16.8	
Single parent	3.6	4.6	
Family type (%)			*p* = .28
Biological parent(s)	98.2	96.1	
Adoptive parent	0.9	0.8	
Step parent	0.9	1.6	
Other	0.0	1.6	

^1^The percentages for the non-participating group do not sum to 100 %, because parents in seven families spend an equal amount of time with their children. The p-value is based on the values shown in the table

#### Randomization

Randomization to the VIPP-Twins or control condition is done at the family level in a ratio of 2:3, using a computer-generated blocked randomization sequence, with a block size of 19 families based on timing of the intervention and stratified by twin gender. Assignment of participants is performed by an independent researcher who is not involved in data collection or coding. Allocation will be performed after the second pretest, right before the start of the intervention, in order to prevent selective attrition. Researchers, interveners and participants are blinded to assignment before, but not after, randomization, because of the open-label design. To minimize bias based on knowledge about allocation of participants, coders and research assistants who carry out the post-intervention home-visits and laboratory sessions are blind to treatment allocation.

### Sample size and power

A meta-analysis on the effects of VIPP-SD on caregiver sensitivity indicates a combined effect size of d = 0.47 [20]. For our primary aim, testing the effect of VIPP-Twins on parental sensitivity and sensitive discipline with a repeated measures analyses with α = .05 and a sample size of 225 families (including 450 children), the power is > 90 % (repeated measures ANOVA within-between interaction, G*Power 3.1.9.2). For our secondary aim (main effects on children’s social competence and behavioral control), the power of the multilevel analysis is > 90 %. For the third and fourth aims, testing moderator effects, the power is > 80 %. For the fifth aim, testing mediating mechanisms, the power is > 90 %.

### Intervention

#### VIPP-SD

The experimental group (40 % of the sample, randomly selected) will receive the Video-feedback Intervention to promote Positive Parenting and Sensitive Discipline - Twins (VIPP-Twins) between the second and third assessment. The VIPP-SD consists of five biweekly sessions in which families are visited at home by a female intervener. All interveners are extensively trained in implementing the intervention by using a standardized manual describing the structure, themes, tips, and exercises for parent and children for each session (manual VIPP-SD version 3.0; [25]). Every session starts with videotaping approximately 15 minutes of standardized parent-child interactions, such as playing or reading a book together [4]. Between sessions, the intervener prepares comments on the child’s or parent’s behavior based on the theme of the next session and selects illustrating video fragments. In the next session, after new video material is collected, the intervener reviews the video of the previous session with the parent and gives video feedback on the chosen video fragments. During this feedback period, the intervener focuses on positive and successful interaction moments and indicates when positive parenting is effective. The parent is explicitly acknowledged as the expert on her own child. The first four intervention sessions each have their own themes with respect to sensitivity and sensitive discipline [19].

The first session focuses on exploration versus attachment behavior, showing the difference between the child’s play and proximity seeking together with the differential parent responses needed, and addresses the use of distraction and inductive discipline as non-coercive responses to difficult child behavior. During the second session, attention is drawn to the perception of the child’s (subtle) signals, using ‘speaking for the child’, and to the use of positive reinforcement by praising positive child behavior and ignoring negative attention seeking. In the third session, the importance of prompt and adequate responding to the child’s signals is explained by showing positive interaction chains between parent and child and the parent is taught to use a sensitive time-out to deescalate temper tantrums. The themes of the fourth session are sharing emotions, showing the parent the importance of attunement in both positive and negative emotions of their child, and promoting empathy for the child during consistent and adequate discipline strategies and clear limit setting. In the first four sessions, only the primary parent is present. The final session is a booster session, in which the different themes are repeated and integrated. The parents’ partner is invited to participate in the final session. Interveners will keep logs about adherence to the intervention protocol.

#### VIPP-Twins

The original version of the intervention (VIPP-SD) has been adapted for the use with twin families in the current study (VIPP-Twins). Instead of only including one target child in the intervention sessions, both twins are included. Parenting a twin may lead to different kinds of challenges for parents, such as dividing attention and sharing or competition between twins, which are less relevant for parents with singletons. To develop the VIPP-Twins protocol, the VIPP-SD was first revised using insights from the VIPP-CC in home-based and center-based child care, because of the shared focus on more than one child [21, 22]. Second, suggestions and feedback from two parents with twins were obtained, in order to understand twin-related parenting challenges and to select appropriate tasks for use with twins. Their suggestions were incorporated in the protocol and intervention manual. Finally, the revised VIPP-Twins protocol was administered by two trained interveners in three pilot families with 5-year-old twins. In accordance with the experiences of the interveners, some of the instructions and toys and puzzles used during the parent-child interactions had to be changed to better fit the situation with two children or the age of the children. For the final VIPP-Twins protocol, we adapted some of the parent-child interaction situations that were videotaped and used for feedback. For example, we included a play situation in which twins have to take turns and one in which the twins are asked to make a puzzle individually and as quickly as possible, in order to create a competitive element.

#### Control condition

Families in the control group will receive six phone calls from a research assistant during the same period as the interventions sessions. This ‘dummy’ intervention will be implemented to ensure the same attention is given to the intervention and control families. During the six protocolized phone calls, parents will be invited to talk about the general development of their twins in a semi-structured interview format. However, they do not receive any specific information or advice about parenting or child development (e.g., [4]).

### Measures

#### Primary outcomes

Our primary aim is to examine the intervention effects on parental sensitivity and sensitive discipline, using several parent-child observations. Parental sensitivity is assessed during free play and structured play situations [26, 27]. Parental discipline is observed during a compliance task, in which the parent is asked to instruct the child to do something he/she does not like (e.g., cleaning up) or to refrain from touching attractive toys [26, 27]. Observations of parental sensitivity and sensitive discipline are performed for the twins separately. In addition, families will receive a video camera to record two evening mealtimes, as naturalistic, daily occurring contexts with intensive family interactions. All tasks are videotaped and coded for parental sensitivity or sensitive discipline by trained coders. Coders will be trained to intercoder reliability ICC > .65, Pearson’s r > .70, and regular meetings and checks will be organized to prevent coder drift. For each construct aggregate measures across ratings and settings will be constructed.

#### Secondary outcomes

The intervention effects on children’s behavioral control and social competence are the secondary outcomes of the study. Both behavioral control and social competence are measured with multiple observational measures as well as questionnaires. Each of the measures will be adjusted to the children’s age at the different time points, and aggregate variables across settings and measures will be constructed, based on factor loadings > .40, for inclusion in analyses. In the case of questionnaires, only scales with internal consistencies > .65 will be included in the analyses.

##### Behavioral control

Children’s ability to control their behavior will be assessed with various observational tasks; a stop-signal task [28], a cheating task [29, 30], and a delay discounting task [31, 32]. Each of these tasks measures children’s ability to inhibit a certain behavior in different situations. Further, a social aggression task will be used to measure children’s aggressive response to acceptance or rejection by peers [33]. Age-adequate adaptations of the measures will be used for the different age groups. In addition, the effortful control scales from the temperament questionnaires will be completed by both parents at each time point and by the children themselves from 7 years of age onward [34, 35].

##### Social competence

Social competence will also be assessed with three different observational tasks and a questionnaire. First, a donating task in which children can donate something they earned (stickers or money, dependent on the child’s age) will be used to measure costly prosocial behavior of the children [9, 36]. Second, in the prosocial Cyberball game, participants have the opportunity to compensate for the exclusion of another player, which is a measure of non-costly prosocial behavior [37]. Again, age-adequate adaptations of the measures will be used for the different age groups. Additionally, both parents will complete the prosocial behavior scale from the Strength and Difficulties Questionnaire (SDQ; [38, 39]) at each time point.

#### Third and fourth aim

##### Susceptibility markers

Our third and fourth aims are to test if the intervention effects on parenting or child outcomes are moderated by parental or children’s genotype or reactive temperament in line with a differential susceptibility model, [40]). Buccal cells will be collected from the children and both parents using Whatman Omniswabs in order to obtain information about genetic polymorphisms of specific dopamine related genes. Parental and children’s reactive temperament will be measured using subscales from Rothbarth’s temperament questionnaires (fearful and reactive temperament, sensory sensitivity; [34, 41]).

#### Fifth aim

##### Neurobiological factors

In addition, we will examine whether the intervention effects on children’s behavioral outcomes are mediated by neurobiological factors. We will collect saliva and hair samples to measure children’s hormonal functioning, with a specific focus on stress hormones (in particular cortisol). Children’s neural (re-)activity will be measured during the previously described social aggression task and prosocial cyberball game, using EEG (focusing on frontal asymmetry) or fMRI (focusing on neural correlates of prosocial behavior and aggression regulation), depending on the age of the child. We will use structural MRI and Diffusion Tensor Imaging (DTI) to measure underlying brain anatomical processes.

### Statistical analyses

Initial data analysis with data inspection steps will be carried out after the research plan and data collection have been finished but before formal statistical analyses are conducted [42]. We will use double data entry for approximately 20 % of the cases and apply range checks for data values, to promote data quality. It will be tested whether missing data are completely at random, at random, or not at random [43], and multiple imputation procedures will be followed to impute missing data. Data transformation will be applied when necessary to approach normal distribution of data points [44]. To avoid any inflation of statistical tests, we are not planning to examine any interim data-sets.

The overall aim of the study is to estimate the effect of the VIPP-Twins. For all aims, the effect of the VIPP-Twins compared to the control condition will be analyzed using intent to treat analyses. For the primary aim, we propose a repeated measures model to estimate the intervention effect on parental sensitivity and sensitive discipline with experimental condition as between subjects factor and assessment time-point as within subjects factor. The regression coefficient of the interaction between condition and time-point estimates differential changes between the intervention and control groups in parental sensitivity and sensitive discipline over time.

Our secondary aim is to examine the effect of the intervention on children’s levels of behavioral control and social competence. In the current study design, twins are nested within families. Depending on the intraclass correlation (ICC) we will use a multilevel approach with three levels (time-point, child, family) or a repeated measures ANOVA with aggregate scores for the twins within families for analyses with child outcomes. Behavioral control and social competence are included in the analyses using aggregated scores across the different measures.

To examine the moderation of the intervention effect, the third and fourth aim of our study, we will include an additional interaction term in the models of aims 1 and 2. For parenting outcomes, we will include the interaction between condition and parental susceptibility markers (genotype and reactive temperament). For child outcomes, we will include an interaction term between parenting and children’s susceptibility markers. For our final aim, exploring mechanisms of intervention effects, we will use the Preacher-Hayes approach [45] in a multilevel or repeated measures design to test for intervention effects on neurobiological variables and examine whether these variables mediate the observed changes in children’s behavioral control and social competence.

### Data management and ethics

Data will be handled strictly confidentially. Data will be stored in the storage environment of the universities Computing Centre in Leiden. Leiden University treats information security in accordance with the International Security Code. Personal information is processed in accordance with the Dutch Personal Information Protection Act which is based on European legislation. The personal data will be handled according to the Dutch Personal Data Protection Act. A separate subject identification code list will be used to link the data and biological specimen to the subject. There will be no personal identification of subjects in scientific communications. We currently do not have ethical permission for data sharing. Access to the final trial dataset will be limited to the formal research team, including principal investigators, post-docs and PhD-students. All members of the research team signed a confidentiality agreement. The L-CID trial is embedded in the larger national Consortium on Individual Development (CID), which unites developmental researchers from seven different universities. For advice on and supervision of the research program, CID composed an international scientific advisory board and a supervisory board to whom our research team reports at least annually.

The research protocol received ethical approval by the Central Committee on Research Involving Human Subjects in the Netherlands (CCMO; NL49069.000.14). Written informed consent for all aspects of the study was obtained from the parents/legal guardians of the twins before the first baseline assessment. Participants were reminded that participating in the trial is voluntary, that they can withdraw from the trial at any time, without consequences and that their data are stored anonymously and securely. All consent forms and related documentation given to the participants were approved by the CCMO and can be requested from the authors. Information for the participants includes the name and contact information of an independent expert (a MD and professor in child and adolescent psychiatry) who will be available during the trial for questions from participants.

The VIPP-SD has been used in twelve previous RCT’s, including in more vulnerable populations [20], and there are no reported risks associated with the intervention. Therefore, there are no criteria for discontinuing the intervention, except on the basis of participants’ own requests. Concomitant care during the trial is not prohibited, but we will use an inventory about previous experiences with video-feedback or other types of preventive care, such as parent training or well-baby clinics. Trial results will be communicated to participants using newsletters about the trial and to professionals in the form of journal articles and scientific conferences. Authorships for journal articles will be determined based on the APA-guidelines and recommendations from the International Committee of Medical Journal Editors. The trial is registered in the Netherlands Trial Registry (NTR; Trial ID: NRT5312, Date registered: July 20, 2015). Any protocol modifications or plans for ancillary studies will be reported to the NTR, CCMO and this journal, and additional informed consent will be obtained from participants.

## Discussion

This study protocol presents the research design of a randomized controlled trial testing the efficacy of a Video-feedback Intervention to promote Positive Parenting and Sensitive Discipline in a twin sample (VIPP-Twins). It is hypothesized that the parenting intervention will enhance parental sensitivity and sensitive discipline strategies and in turn, positively affect children’s developmental outcomes, specifically social competence and behavioral control. In addition, we aim to test the differential susceptibility theory to examine differential efficacy of the intervention based on genetic make-up or temperament for both parents and children. We expect that parents and children with susceptible genotypes [6] or reactive temperaments will profit most from the intervention.

### Strengths and limitations

An important strength of the study is that the intervention program VIPP-SD has a sound theoretical basis in attachment theory as well as coercion theory [19]. In addition, several previous randomized trials have found empirical evidence for the efficacy of the original version of the VIPP and for several adaptations of the program [20]. Therefore, we have a clear hypothesis about the efficacy of the program, and we can now fill in the gap that exists in knowledge of moderating and mediating factors in the program’s effectiveness.

The random assignment of families to the experimental or control condition is the most important strength of the study design. Random assignment of families to the experimental condition reduces potential gene-environment correlations, and opens the way to test the true moderating effect of participants’ characteristics on intervention efficacy. Because of the longitudinal design of the study with multiple follow-up measures, we can test both short-term and long-term effects of the intervention program. Intervention effects are not only measured on the behavioral level, but also on the hormonal and neural level. Information about intervention effects on neurobiological levels of functioning may explain the long lasting intervention behavioral effects that have previously been documented [10]. A final strength of the study concerns the observational and ambulatory measures, which reduce possible reporter bias related to self-report questionnaire studies.

The study has some limitations that should be noted. First, the large sample size demands multiple interveners, which may lead to divergences in program implementation. However, given the extensive training of interveners, the use of a standardized manual, and frequent supervision, we expect high treatment fidelity. A possible limitation in examining differential susceptibility to intervention effects and the mediators for change in child outcomes is the twin sample. Twin families may be different from families with singletons on several aspects, which may decrease the generalizability of the findings (but see: [46, 47]). The twin sample however also provides the opportunity to test for differential susceptibility within families and for genetic modelling of intervention effects.

In conclusion, the current study will evaluate the effects of a video-feedback intervention in a preschooler twin sample. Results of the study will provide insights in the efficacy of the VIPP-Twins and possible moderators and mediators of program efficacy resulting in an experimental test of the differential susceptibility theory.

## Abbreviations

DTI, diffusion tensor imaging; L-CID, Leiden consortium on individual development; RCT, Randomized controlled trial; SDQ, Strengths and difficulties questionnaire; VIPP-CC, Video-feedback intervention to promote positive parenting – child care; VIPP-SD, Video-feedback intervention to promote positive parenting and sensitive discipline; VIPP-Twins, Video-feedback intervention to promote positive parenting – twin families.

## Additional file

Additional file 1: SPIRIT Checklist. (DOC 121 kb)
